# Can Automated Hematology Analyzers Predict the Presence of a Genetic Hemoglobinopathy? An Analysis of Hematological Biomarkers in Cambodian Women

**DOI:** 10.3390/diagnostics11020228

**Published:** 2021-02-03

**Authors:** Lulu X. Pei, Tebogo T. Leepile, Kelsey M. Cochrane, Kaitlyn L. I. Samson, Jordie A. J. Fischer, Brock A. Williams, Hou Kroeun, Lizl Bonifacio, Crystal D. Karakochuk

**Affiliations:** 1 Food, Nutrition and Health, The University of British Columbia, Vancouver, BC V6T 1Z4, Canada; lulu.pei@stat.ubc.ca (L.X.P.); kelsey.cochrane@ubc.ca (K.M.C.); kaitlyn.samson@ubc.ca (K.L.I.S.); jordie.fischer@ubc.ca (J.A.J.F.); brock.williams@ubc.ca (B.A.W.); lizl_bonifacio@hotmail.com (L.B.); 2 Department of Statistics, The University of British Columbia, Vancouver, BC V6T 1Z4, Canada; 3 Integrated Studies in Land and Food Systems, The University of British Columbia, Vancouver, BC V6T 1Z4, Canada; tebogo.leepile@ubc.ca; 4 BC Children’s Hospital Research Institute, Vancouver, BC V5Z 4H4, Canada; 5 Helen Keller International, Phnom Penh, Cambodia; hkroeun@hki.org

**Keywords:** anemia, hemoglobinopathy, hematology analyzer, laboratory, method

## Abstract

Genetic hemoglobinopathies are the most common single-gene disorder worldwide. Some automated hematology analyzers have the capability of flagging individuals who may have hematological disorders based on complete blood count (CBC) biomarkers. We aimed to evaluate the accuracy of a hematology analyzer in identifying genetic hemoglobinopathies in Cambodian women and to determine which hematological biomarkers are the best predictors. A CBC was completed using a Sysmex XN-1000 analyzer and hemoglobinopathies were determined with capillary hemoglobin electrophoresis for 808 nonpregnant Cambodian women. Sysmex XN-1000 Interpretive Program (IP) messages, which flag potential hematological disorders, were produced from CBC results. Then, 2 × 2 tables were used to determine sensitivity and specificity of the IP message “Hemoglobin defect” to detect a genetic hemoglobinopathy. Receiver operating characteristic (ROC) analyses assessed the diagnostic ability of six CBC biomarkers to predict a genetic hemoglobinopathy. In total, 74% of women had a hemoglobinopathy (predominantly Hb E and α-thalassemia). “Hb defect” IP message sensitivity and specificity for genetic hemoglobinopathy detection were 10.4% and 98.6%, respectively. Variable selection strategies yielded a two-variable model including mean corpuscular volume (MCV) and red blood cell (RBC) count (AIC = 99.83, AUC^ROC^ = 0.98 (95% CI: 0.97, 0.99)) for the prediction of a homozygous EE disorder. Sensitivity and specificity values do not justify the use of Sysmex XN-1000 IP flag messages for identification of genetic hemoglobinopathies in Cambodian women. Development of an algorithm based on MCV and RBC biomarkers may optimize the screening ability of automated hematology analyzers.

## 1. Introduction

Genetic hemoglobinopathies are autosomal recessive disorders that result from nucleotide substitutions (e.g., sickle cell or hemoglobin E) or deletions (e.g., α- or β-thalassemia) in the genes encoding the α- or β-globin chains of hemoglobin [1]. Some of these genetic disorders are thought to confer protection against malaria, which is a reason for the high prevalence among historically malaria-endemic regions in Africa and Asia [2,3]. For example, in Cambodia, genetic hemoglobinopathies affect approximately half of the population (most common are the hemoglobin E disorder and α-thalassemia) [4,5,6]. These hemoglobinopathies can result in decreased or defective hemoglobin production, leading to an increased risk of anemia and other serious health problems [1,7]. Clinical outcomes typically depend on the severity of the disorder. Individuals who inherit only one affected allele are termed heterozygous (also referred to as traits), whereas individuals who inherit two affected alleles are termed homozygous, which typically results in a more severe phenotype (e.g., severe anemia).

Genetic hemoglobinopathies can be detected using several methods such as capillary hemoglobin electrophoresis, isoelectric focusing, or high-performance liquid chromatography; however, DNA analysis is sometimes required for definitive diagnosis [8]. These laboratory tests can be complicated and expensive; thus, they are not routinely conducted in rural areas and/or low-resource settings. Despite this, the early diagnosis of genetic hemoglobinopathies is a high priority in order to provide those at risk with adequate care and prevent exacerbation of the disease and its comorbidities.

Automated hematology analyzers are blood count analyzers that are capable of measuring numerous hematological biomarkers, also referred to as a complete blood count (CBC), in a small blood sample. The biomarkers included in a CBC vary by analyzer but often include hemoglobin, hematocrit, red blood cells (RBCs), white blood cells (WBCs), mean corpuscular volume (MCV), mean corpuscular hemoglobin (MCH), and red blood cell distribution width (RDW). These biomarkers are often used to diagnose and categorize anemia type and severity and to assess for other hematological disorders. The Sysmex XN-1000 is a hematology analyzer which generates a “flag” for individuals who may be at risk of certain hematological disorders based on the CBC results (referred to as an interpretive program (IP) message). Some examples of Sysmex XN-1000 IP messages include: RBC agglutination (clumping), iron deficiency, and hemoglobin defects [9]. IP messages are based on proprietary algorithms which consider a combination of various hematological items analyzed (e.g., suspect hemoglobin defect is determined by calculation and size comparison of MCV and RDW-CV). The accuracy of the Sysmex analyzer to correctly “flag” individuals at high risk of a genetic hemoglobin defect is of critical importance, as this information could be used to refer these high-risk individuals for more definitive testing (which can be expensive and difficult to access). This would lead to more accurate diagnoses and medical follow up, which would significantly improve the health of individuals living with these conditions.

The aims of this study were to evaluate the accuracy of the IP message for hemoglobin defects (“Hb defect”) in identifying Cambodian women with a genetic hemoglobinopathy by comparing these flags to definitive testing performed through hemoglobin electrophoresis and to determine which combination of CBC biomarkers can best predict the presence of a genetic hemoglobinopathy.

## 2. Materials and Methods

Data used in this analysis were obtained from a previously conducted randomized controlled trial of oral iron supplementation among 809 nonpregnant Cambodian women in 2015. Ethical approval was obtained from the Clinical Research Ethics Board at the University of British Columbia, Canada (H15-00933, 19 June 2015) and the National Ethics Committee for Health Research, Cambodia (110-NECHR, 24 April 2015) and was registered at ClinicalTrials.gov (NCT02481375). The full trial has been reported elsewhere [10]. Study inclusion criteria included healthy, nonpregnant women between the ages of 18-45 years with a hemoglobin concentration < 117 g/L based on a capillary finger-prick blood sample using a HemoCue Hb 301 hemoglobinometer (HemoCue AB, Angelholm, Sweden).

We used data previously collected from the 2015 trial, including baseline CBC biomarkers and genetic hemoglobin typing, to determine the presence of a genetic hemoglobinopathy. Fasting venous blood was collected in a 2 mL tube containing EDTA (Becton Dickinson, Franklin Lakes, NJ, USA) at baseline and after 12 weeks of supplementation and transported on ice within 2–4 h of collection to the National Institute of Public Health Laboratory (NIPHL) in Phnom Penh for processing. A CBC was performed using the Sysmex XN-1000 automated hematology analyzer (Sysmex Corporation, Kobe, Japan) to measure hemoglobin (g/dL), reticulocyte hemoglobin (g/L), MCV (fL), MCH (pg), and RDW (%).

DNA was extracted from buffy coat by using a QiaAmp Blood DNA kit (Qiagen Ltd., Hilden, Germany) and assessed for the following 21 α-globin gene deletions and point mutations with the use of the α-globin StripAssay kit (ViennaLab Diagnostics, Vienna, Austria): 3.7 α-gene deletion, 4.2 α-gene deletion, MED double α-gene deletion, SEA double α-gene deletion, THAI double α-gene deletion, FIL double α-gene deletion, 20.5 kb double α-gene deletion, anti-3.7 α-gene triplication, α1 cd 14 [TGG>TAG], α1 cd 59 [GGC>GAC] (Hb Adana), α2 init cd [ATG>ACG], α2 cd 19 [-G], α2 IVS1 [-5nt], α2 cd 59 [GGC>GAC], α2 cd 125 [CTG>CCG] (Hb Quong Sze), α2 cd 142 [TAA>CAA] (Hb Constant Spring), α2 cd 142 [TAA>AAA] (Hb Icaria), α2 cd 142 [TAA>TAT] (Hb Pakse), α2 cd 142 [TAA>TCA] (Hb Koya Dora), α2 poly A-1 [AATAAA-AATAAG], α2 poly A-2 [AATAAA-AATGAA].

Capillary hemoglobin electrophoresis for the detection of hemoglobin variants (E, CS, H, Bart, or F) was conducted by a trained external consultant at NIPHL in Cambodia using a MINICAP analyzer (Sebia, Lisses, France). This automated technique quantifies the different types of Hb in blood for interpretive diagnosis and can detect normal Hb (Hb A, A2, and F) and Hb variants (Hb E, H, and Constant Spring (CS)). The Sebia analyzer has an advantage over other methodologies as it can distinguish between Hb A2 and Hb E variants, both of which are common in Cambodia [11]. Hemoglobin E variants are described either as in heterozygous (“AE”) or homozygous (“EE”) form.

The primary outcome for this analysis was the presence of a genetic hemoglobinopathy, previously determined using genetic hemoglobin typing conducted as part of the original 2015 trial [10]. The “Hb defect” Sysmex IP flag message was investigated for its ability to accurately identify a woman with a genetic hemoglobinopathy. We also investigated the ability of any Sysmex IP flag message (including iron deficiency, abnormal platelet distribution, platelet clumps, nucleated RBCs present, microcytosis, anisocytosis, anemia, fragments, and abnormal reticulocyte scattergram) to accurately identify a woman with a genetic hemoglobinopathy.

CBC biomarkers (hemoglobin, reticulocyte hemoglobin, MCV, MCH, RDW, and RBC) were compared across groups of women with a genetic hemoglobinopathy and those without, using Kruskal–Wallis tests with subsequent posthoc evaluation of pairwise comparisons. Sensitivity and specificity values were calculated from 2 × 2 contingency tables as an initial assessment of the accuracy of the IP flag messages in correctly identifying women with a genetic hemoglobinopathy. Receiver operating characteristic (ROC) analyses were used to assess the diagnostic ability of the six CBC biomarkers to predict whether or not a woman had a genetic hemoglobinopathy, with the area under the ROC curve (AUC^ROC^) serving as a measure of the ability of the model to discriminate between women likely to have a hemoglobinopathy and those unlikely to have a hemoglobinopathy. AUC^ROC^ values range from 0.5 to 1, where larger values indicate greater discrimination ability and stronger predictive power; generally, an AUC^ROC^ value greater than 0.8 denotes a good classifier [12].

A multivariate logistic model was used to assess the joint predictive ability of multiple CBC biomarkers for the most severe hemoglobinopathy: the homozygous EE disorder. ROC analyses were also used to evaluate the discrimination performance of the CBC biomarkers to predict if a woman had the homozygous EE disorder. To obtain the multibiomarker predictive model, variable selection was performed using backwards elimination while taking into consideration potential multicollinearity and the variance inflation factor (VIF) and correlation values between predictors. Univariate (unadjusted) analysis through fitting single-biomarker models was also performed to determine the directionality of the association between the CBC hematological biomarkers and the outcome of interest. Both variable significance and Akaike information criterion (AIC) were considered in the process of selection. Further assessment of model predictive ability was performed through cross-validation methods of splitting the observed data into training and validation sets in order to flag problems such as overfitting or selection bias [13].

Statistical analyses were conducted using R version 3.6.3 (R Core Team 2020) and SAS version 9.4 (SAS Inc., Cary, NC, USA).

## 3. Results

### 3.1. Study Population Characteristics

Data from a total of *n* = 808 women (99%) from the original trial were available for these diagnostic analyses; hematological values for *n* = 1 woman were missing due to a clotted sample and the subsequent inability to perform a CBC. Characteristics and hematological statuses of enrolled women are presented in Table 1.

Table 2 summarizes the distributions of CBC biomarkers in women with no hemoglobinopathy compared with women with specific hemoglobinopathies (α-thalassemia, heterozygous AE, coinherited α-thalassemia/heterozygous AE, and homozygous EE). Women were included in the “α-thalassemia” group if they had any of the α-gene point mutation or deletions. Women were included in the “homozygous EE” group if they had the severe disorder, with or without coinheritance of any other disorder, due to rarity and severity of this disorder. Kruskal–Wallis nonparametric tests were conducted on the distributions of biomarkers across hemoglobinopathy classification groups (*p* < 0.0001, adjusted for multiple comparisons). Posthoc Dunn’s multiple comparison tests were performed across each biomarker to compare distributions between two selected hemoglobinopathy classification groups.

Notably, women with the homozygous EE disorder had significantly different distributions (*p* < 0.0001) for all biomarkers as compared with both women with no disorder and compared with women with any of the other hemoglobinopathies (α-thalassemia, heterozygous AE, and coinherited α-thalassemia/heterozygous AE). Biomarker distributions between α-thalassemia, heterozygous AE, and coinherited α-thalassemia/heterozygous AE groups were not significantly different for any biomarker except for MCV where there was a significant difference between α-thalassemia and heterozygous AE groups (*p* = 0.0009). Comparisons between the “no disorder” group and the other specific hemoglobinopathies (aside from homozygous EE) were significant for reticulocyte hemoglobin, MCV, MCH, RDW, and RBC (*p* < 0.0001)—these significant comparisons were not observed for hemoglobin.

We also compared the distributions of CBC biomarkers in women with no hemoglobinopathy to women with “any” hemoglobinopathy (as a combined group, including women with other rare disorders such as Hb CS and F) (Table S1). Notably, significant differences were observed for all biomarkers between these two groups (Wilcoxon rank-sum nonparametric tests; *p* < 0.0001).

### 3.2. IP Message Assessment

Assessment of the sensitivity and specificity of the Sysmex IP message (“Hb defect”) was performed through calculation from 2 × 2 tables displaying the agreement between the IP flag and the presence of a true genetic hemoglobinopathy. The performance of the “Hb defect” flag alone was considered in addition to the performance of any IP message at flagging women with genetic hemoglobinopathies. Sensitivity and specificity values of these flag messages are summarized in Table 3.

### 3.3. ROC Analyses

**Figure 1:**
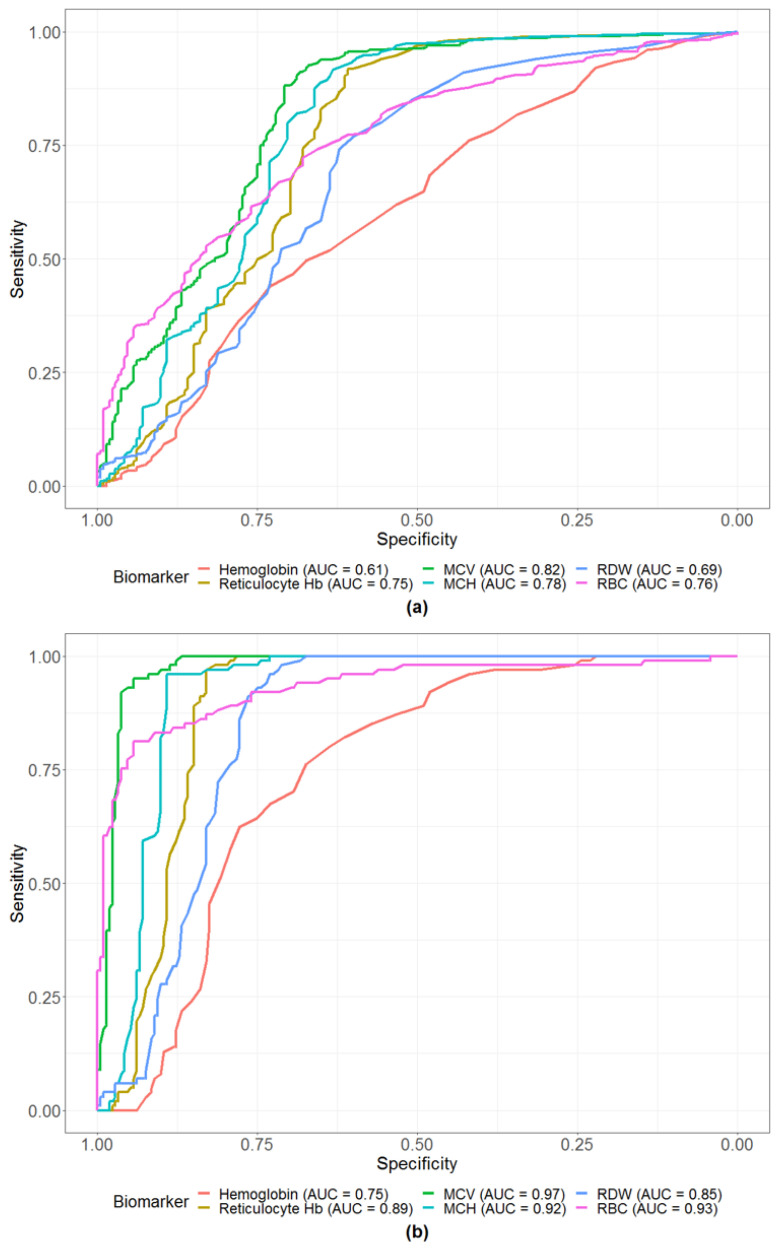
(**a**) Receiver operating characteristic (ROC) curves for the individual CBC biomarkers in the prediction of any genetic hemoglobinopathy. (**b**) Receiver operating characteristic (ROC) curves for the individual CBC biomarkers in the prediction of the homozygous EE disorder. AUC, area under the curve; Hb, hemoglobin; MCV, mean corpuscular volume; MCH, mean corpuscular hemoglobin; RDW, red blood cell distribution width; RBC, red blood cell.

ROC curves and corresponding AUC^ROC^ values of the six CBC biomarkers for predicting (a) any genetic hemoglobinopathy and (b) the homozygous EE disorder are displayed in Figure 1. Additional ROC curves for predicting α-thalassemia, heterozygous AE, and coinherited α-thalassemia/heterozygous AE can be found in Figures S1–S3, respectively.

### 3.4. Multibiomarker Predictive Model

Individual CBC biomarkers appeared to perform very well in terms of predicting the homozygous EE disorder (with five out of the six chosen biomarkers having AUC^ROC^ values > 0.8). However, further exploration of potential improvements in predictive ability through the simultaneous evaluation of several biomarkers may justify a reliable multibiomarker algorithm. Among the six biomarkers, only MCV and RBC retained the proper directionality once included in the multivariate model, suggesting that multicollinearity is present for the other biomarkers. VIF values for the remaining biomarkers were also found to be large (>10), further justifying their exclusion.

Variable selection strategies yielded a two-variable model including MCV and RBC (AIC = 99.83, AUC^ROC^ = 0.98 (95% CI: 0.97, 0.99)). Performing a likelihood ratio test between this multibiomarker model and a model containing MCV alone (AIC = 107.34, AUC^ROC^ = 0.97 (95% CI: 0.96, 0.99)) revealed a significant result (*p* = 0.002) in favor of the multibiomarker model in terms of model fit. The regression summary of the final multivariate model is presented in Table 4. Including both MCV and RDW biomarkers in the same model artificially alters the directionality of their association with presence of a homozygous EE disorder. This change in the signs of regression coefficients is evidence of multicollinearity and indicates that MCV and RDW should not be included together in the same predictive model.

Performing cross-validation on the multibiomarker model using training set and validation set splits of 50:50, 70:30, and 80:20 revealed insignificant AUC^ROC^ comparisons (*p* = 0.989, *p* = 0.479, *p* = 0.096, respectively, obtained from asymptotic chi-square distributions) with all AUC^ROC^ values > 0.98. This suggests good robust predictive performance of the proposed model with biomarkers of MCV and RBC.

The multibiomarker model summarized in Table 4 serves as a tool for estimating a woman’s expected probability of having the homozygous EE disorder. The regression summary suggests that lower MCV and higher RBC count are associated with an increased risk of having the EE disorder.

Predicted probabilities of having a hemoglobinopathy can be calculated from the risk scores (log odds ratios) given by the regression equation for the selected predictive model:

risk score = 12.76 − 0.31(MCV) + 1.58(RBC)(1)

The predicted probability of having the homozygous EE disorder can be calculated as:

*e*^risk score^/(1 + *e*^risk score^) (2)

The predicted probability obtained from a woman’s baseline MCV and RBC measurements can then be used for screening purposes. A higher value of the risk score suggests a greater risk of the woman having the homozygous EE disorder. In screening, it may be desired to select a cut-off to identify women that are likely to have the disorder. A predicted probability of 0.5 can be applied as a conservative cut-off; however, field-specific knowledge can further guide the selection of this threshold value. Predicted probabilities higher than this cut-off value suggest an individual is at high risk of having the homozygous EE disorder, and as such, it may be beneficial to recommend the individual for further definitive testing.

## 4. Discussion

In our study among Cambodian women of reproductive age, we found that the Sysmex XN-1000 IP message “Hb defect” was not accurate in flagging women with a genetic hemoglobinopathy. In fact, women who were flagged for any IP message (generic) had a higher sensitivity and specificity trade-off as compared with women who were flagged for the “Hb defect” (specific). Ultimately, the sensitivity and specificity values (for any of the IP messages) are not high enough to justify using the Sysmex IP flag messages as a preliminary screen for the presence of a genetic hemoglobinopathy. With such low sensitivity, too many cases (i.e., women with a true genetic hemoglobinopathy) would likely be missed.

The “Hb defect” flag is purportedly obtained with use of an algorithm that is based upon MCV and RDW values. We observed that including both MCV and RDW biomarkers in the same model for predicting the presence of a homozygous EE disorder artificially altered the directionality of their association with the outcome. This change in sign of regression coefficients is evidence of multicollinearity and indicates that MCV and RDW should not be included together in the same predictive model. As such, the current algorithm may not be appropriate for flagging this severe form of genetic disorder. Across both sets of ROC curves, MCV consistently appeared to have the strongest predictive ability of all six biomarkers. Therefore, if only one biomarker should be included in an algorithm designed to predict hemoglobinopathies, our data would suggest MCV over RDW.

The “Hb defect” flag performed poorly when it was specifically used to detect a homozygous EE disorder, as compared with when it was used to detect “any” hemoglobinopathy, as well as when it was used to detect any of the other hemoglobinopathies (α-thalassemia, heterozygous AE, and coinherited α-thalassemia/heterozygous AE). AUC^ROC^ values for all CBC biomarkers, however, were higher in the homozygous EE disorder model, as compared with the “any” hemoglobinopathy model. The homozygous EE disorder is a severe disorder which has a more clinically distinct phenotype (e.g., more severe anemia), suggesting that development of an accurate IP flag for this specific disorder may be more clinically relevant and appropriate. Individuals flagged for this severe disorder could then be referred for further definitive genetic testing, which would result in better targeting for follow-up testing.

In 2015, Hoffmann et al. conducted a meta-analysis of the 12 indices most frequently used to differentiate iron deficiency anemia from thalassemia trait among those with microcytic anemia [14]. Here, the authors found that the ratio of microcytic RBC%/hypochromic RBC% performed better as a differential diagnosis of microcytic anemia than other discriminant indices. The meta-analysis showed that population age (adult or child) and geographical region were both important factors in determining the diagnostic usefulness of the indices when distinguishing thalassemia from iron deficiency anemia; indices generally performed better in adults, compared with children, and the highest diagnostic odds ratio was found in studies from Europe, while the lowest diagnostic odds ratio was found in Southeast Asian populations. As our population is from Cambodia, the proposed microcytic RBC%/hypochromic RBC% ratio may not be an appropriate diagnostic tool.

Nivaggioni et al. analyzed hematological parameters from the Sysmex XN-10 (a different analyzer than the Sysmex XN-1000) using a two-step decision by classification and regression tree (CART) to predict types of anemia, including iron deficiency anemia, heterozygous haemoglobinopathy, sickle cell disease syndrome, hereditary spherocytosis and other, in 8217 patients (15+ years) from a hospital in France [15]. Here, the CART method found that the percentage of microcytes, RDW, percentage of nucleated red blood cells, MCH, and immature reticulocyte fraction resulted in a correct disorder classification rate of 99.4%. It should be noted that as these CBCs were performed on the Sysmex XN-10, this classification algorithm may not be applicable to other automated hematology analyzers. The success of these parameters in this population, in contrast to our results and those of the meta-analysis above, suggests that region-specific algorithms are likely needed to help screen for genetic hemoglobinopathies.

Karnpean et al. have also showed that the sensitivity of MCV and MCH biomarkers to predict a hemoglobin E disorder is substantially increased when assessed in combination with a simple dye test (dichlorophenolindophenol or DCIP test) [16]. The authors found that using a cut-offs of 78 fL and 27 pg for MCV and MCH, respectively, in combination with the DCIP test revealed 100% sensitivity and ~80% specificity in the screening of α-thalassemia, ß-thalassemia and Hb E in 279 Southeast Asian individuals [16]. Administering the DCIP test would require additional resources (time and cost), but would result in improved screening, especially for Hb E disorders which are common in this region of the world.

A strength of our study is our comprehensive genotyping for the most common hemoglobinopathies in Cambodian women (Hb variants (E, H, CS) and 21 different α-globin deletions and point mutations); however, although comprehensive, we recognize that this list may not be exhaustive. Other rare hemoglobinopathies may have been present in women but were not captured by our comprehensive genomic testing. Further, we acknowledge that the “Hb defect” IP flag may be more accurate in identifying other hemoglobinopathies that are more common in other geographical areas of the world, such as sickle cell disease in sub-Saharan Africa [3]. We based our analysis on the premise that this flag would have the ability to detect a genetic hemoglobinopathy, but it could, in fact, be more appropriate to detect other “Hb defects” that we did not investigate, such as hemolytic disorders. Lastly, performance of predictive models is inherently overestimated: we used cross-validation techniques to assess and reduce this risk (which was deemed minimal), but confirmation of the performance of our predictive model would still be recommended in future data sets.

## Figures and Tables

**Table 1:** Characteristics of all enrolled Cambodian women (18–45 years).

	*n* = 809 Women
Age, years	30 (23, 35)
Household size, *n*	4.8 ± 1.7
**Completed education level, *n* (%)**	
None	104 (13%)
Primary	450 (56%)
Lower Secondary	218 (27%)
Upper Secondary	36 (4%)
Higher education/University	1 (<1%)
**Parity**	
0	(29%)
1–2	306 (38%)
3+	268 (33%)
Hemoglobin, g/dL	11.7 (10.9, 12.5)
Anemia, hemoglobin < 12.0 g/dL, *n*/total (%)	468/808 (58%)

Values are mean ± SD, median (IQR), or *n* (%). For hemoglobin concentration and anemia prevalence; *n* = 1 missing due to a clotted sample.

**Table 2:** Distributions of complete blood count (CBC) biomarkers among women, by hemoglobinopathy classification.

	No Hemoglobin-opathy	Genetic Hemoglobinopathies			
		α-Thalassemia	Heterozygous AE	Coinherited α-Thalassemia and Heterozygous AE	Homozygous EE
*n* (%)	212/808 (26%)	171/808 (21%)	186/808 (23%)	133/808 (16%)	101/808 (13%)
Hemoglobin, g/dL	12.0 (11.3, 12.8) ^a^	11.8 (10.8, 12.4) ^a^	11.8 (11.0, 12.5) ^a^	11.8 (10.7, 12.4) ^a^	10.9 (10.5, 11.5) ^b^
Reticulocyte hemoglobin, g/L	29.6 (25.9, 30.6) ^a^	26.3 (23.3, 28.2) ^b^	26.5 (25.4, 27.3) ^b^	26.8 (23.9, 28.0) ^b^	21.8 (20.6, 22.7) ^b^
MCV, fL	85.9 (79.3, 89.6) ^a^	79.2 (71.4, 82.9) ^b^	76.7 (73.7, 79.0) ^b^	77.3 (72.2, 81.6) ^b^	61 (59.2, 62.7) ^b^
MCH, pg	28.3 (25.0, 29.4) ^a^	24.8 (21.9, 26.6) ^b^	24.9 (23.9, 25.9) ^b^	25.1 (22.8, 26.7) ^b^	20.2 (19.3, 21.0) ^b^
RDW, %	13.1 (12.4, 14.9) ^a^	14.0 (13.2, 15.4) ^b^	14.2 (13.5, 14.8) ^b^	13.9 (13.2, 15.4) ^b^	16.6 (15.8, 17.8) ^b^
RBC, 10^12^/L	4.39 (4.16, 4.71) ^a^	4.79 (4.49, 5.12) ^b^	4.79 (4.55, 5.05) ^b^	4.76 (4.49, 5.09) ^b^	5.52 (5.23, 5.75) ^b^

Values are median (IQR). CBC, complete blood count; MCV, mean corpuscular volume; MCH, mean corpuscular hemoglobin; RDW, red blood cell distribution width; RBC, red blood cell. Values with different superscript letters (a or b) in each row indicate significant differences between women with each of the specific hemoglobinopathies compared to women with no hemoglobinopathy (Dunn’s multiple pairwise comparisons, *p* < 0.0001).

**Table 3:** Sensitivity and specificity values classified by women who were flagged for the “Hb defect” IP message and by women who were flagged by any IP message classification.

	Any Hb Disorder		α-Thalassemia		Heterozygous AE		Coinherited α-Thalassemia and Heterozygous AE		Homozygous EE	
	Hb Defect	Any IP	Hb Defect	Any IP	Hb Defect	Any IP	Hb Defect	Any IP	Hb Defect	Any IP
Sensitivity	10.4%	41.1%	8.8%	33.9%	14.0%	30.1%	10.5%	35.3%	6.9%	81.2%
Specificity	98.6%	82.1%	98.6%	82.1%	98.6%	82.1%	98.6%	82.1%	98.6%	82.1%

Hb, hemoglobin; IP, interpretive program.

**Table 4:** Regression model summary of the multibiomarker model for predicting presence of the homozygous EE disorder.

	Coefficient (95% CI)	SE	*p*-Value	OR (95% CI)
MCV, fL	−0.31 (−0.41, −0.21)	0.05	<0.001	0.74 (0.67, 0.81)
RBC, 10^12^/L	1.58 (0.55, 2.60)	0.52	0.003	4.85 (1.74, 13.50)
Intercept	12.76 (4.03, 21.49)	4.45	0.004	NA

Values are regression coefficients (exponentiated to obtain odds ratios), presented with 95% CIs. Multivariate predictive model obtained through consideration of multicollinearity and correlations between CBC biomarkers. CBC, complete blood count; CI, confidence interval; MCV, mean corpuscular volume; OR, odds ratio; RBC, red blood cell; SE, standard error.

## Data Availability

The data presented in this study are available on request from the corresponding author.

## Supplementary Materials

The following are available online at https://www.mdpi.com/2075-4418/11/2/228/s1, Table S1: Distributions of CBC biomarkers among women, by presence or absence of a genetic hemoglobinopathy; Figure S1: Receiver operating characteristic (ROC) curves for the individual CBC biomarkers in the prediction of α-thalassemia; Figure S2: Receiver operating characteristic (ROC) curves for the individual CBC biomarkers in the prediction of the heterozygous AE disorder; Figure S3: Receiver operating characteristic (ROC) curves for the individual CBC biomarkers in the prediction of coinherited heterozygous AE disorder and α-thalassemia.
