# Supplementary material for: Can Automated Hematology Analyzers Predict the Presence of a Genetic Hemoglobinopathy? An Analysis of Hematological Biomarkers in Cambodian Women
Source: Diagnostics (Basel). 2021 Feb 3;11(2):228. doi: 10.3390/diagnostics11020228 (PMC7913495; doi:10.3390/diagnostics11020228)
Supplement: Supplementary file 1 [file diagnostics-11-00228-s001.pdf]

diagnostics

MDPI

Supplementary File

Supplementary Tables

Table S1. Distributions of CBC biomarkers among women, by presence or absence of a genetic hemoglobinopathy.

	No Hemoglobinopathy	Any Hemoglobinopathy [1]
n (%)	212/808 (26%)	596/808 (74%)
Hemoglobin, g/dL	12.0 (11.3, 12.8) [a]	11.6 (10.7, 12.3) [b]
Reticulocyte hemoglobin, g/L	29.6 (25.9, 30.6) [a]	25.9 (22.8, 27.5) [b]
MCV, fL	85.9 (79.3, 89.6) [a]	76 (67.4, 80.2) [b]
MCH, pg	28.3 (25.0, 29.4) [a]	24.3 (21.2, 26.0) [b]
RDW, %	13.1 (12.4, 14.9) [a]	14.4 (13.4, 16.2) [b]
RBC, 10^{12}/L	4.39 (4.16, 4.71) [a]	4.87 (4.57, 5.23) [b]

Values are median (IQR). CBC, complete blood count; MCV, mean corpuscular volume; MCH, mean corpuscular hemoglobin; RDW, red cell distribution width; RBC, red blood cell. Comparisons between no disorder and any disorder groups significant for all biomarkers (Wilcoxon rank-sum tests, $p < 0.0001$) as indicated by different superscript letters (a or b) in each row. [1] Any hemoglobinopathy includes $n = 5$ women who had a genetic Hb disorder but not one of α-thalassemia, heterozygous AE or homozygous EE.

Supplementary Figures

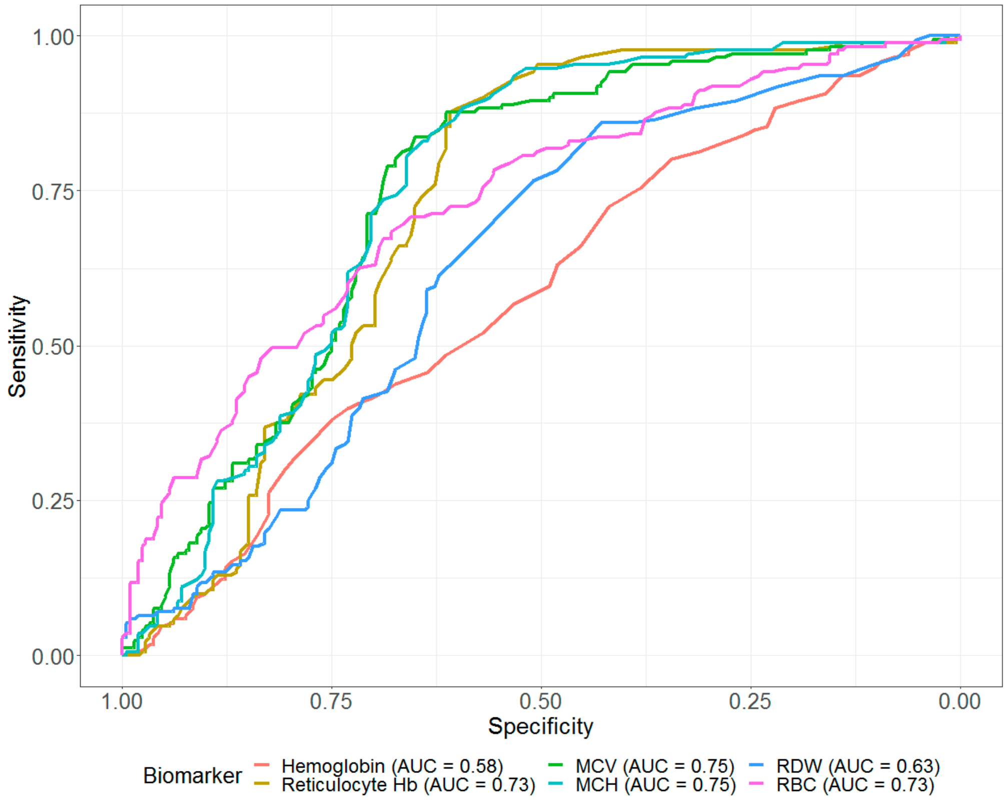

Figure S1. Receiver operating characteristic (ROC) curves for the individual CBC biomarkers in the prediction of α-thalassemia. AUC, area under the curve; Hb, hemoglobin; MCV, mean corpuscular volume; MCH, mean corpuscular hemoglobin; RDW, red blood cell distribution width; RBC, red blood cell.

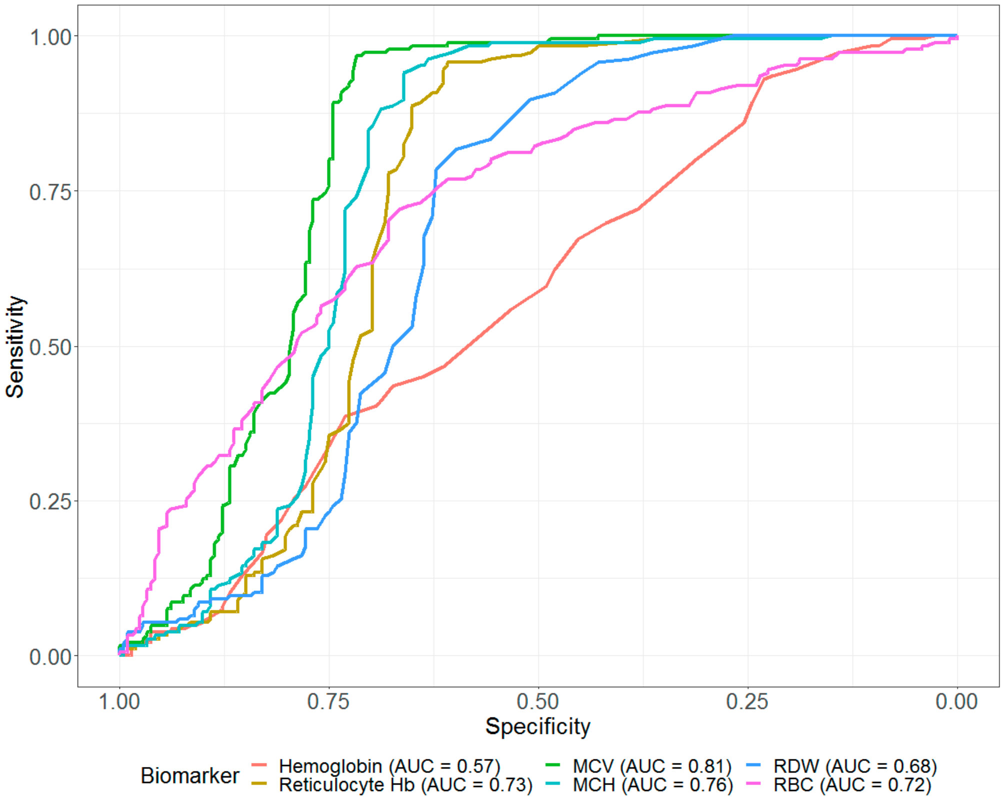

Figure S2. Receiver operating characteristic (ROC) curves for the individual CBC biomarkers in the prediction of the heterozygous AE disorder. AUC, area under the curve; Hb, hemoglobin; MCV, mean corpuscular volume; MCH, mean corpuscular hemoglobin; RDW, red blood cell distribution width; RBC, red blood cell.

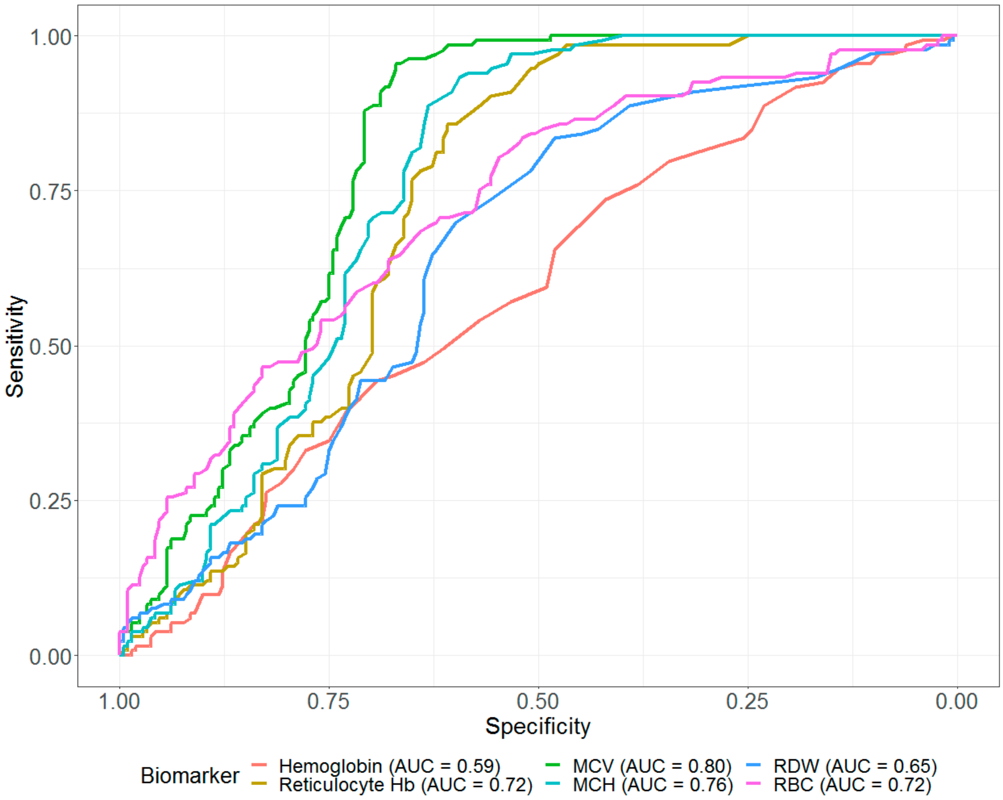

Figure S3. Receiver operating characteristic (ROC) curves for the individual CBC biomarkers in the prediction of co-inherited heterozygous AE disorder and α-thalassemia. AUC, area under the curve; Hb, hemoglobin; MCV, mean corpuscular volume; MCH, mean corpuscular hemoglobin; RDW, red blood cell distribution width; RBC, red blood cell.
